# Social contact patterns relevant to the spread of respiratory infectious diseases in Hong Kong

**DOI:** 10.1038/s41598-017-08241-1

**Published:** 2017-08-11

**Authors:** Kathy Leung, Mark Jit, Eric H. Y. Lau, Joseph T. Wu

**Affiliations:** 1 WHO Collaborating Centre for Infectious Disease Epidemiology and Control, School of Public Health, Li Ka Shing Faculty of Medicine, The University of Hong Kong, Hong Kong SAR, People’s Republic of China; 2 Department of Infectious Disease Epidemiology, London School of Hygiene and Tropical Medicine, London, United Kingdom; 3 Modelling and Economics Unit, Public Health England, London, United Kingdom

## Abstract

The spread of many respiratory infections is determined by contact patterns between infectious and susceptible individuals in the population. There are no published data for quantifying social contact patterns relevant to the spread of respiratory infectious diseases in Hong Kong which is a hotspot for emerging infectious diseases due to its high population density and connectivity in the air transportation network. We adopted a commonly used diary-based design to conduct a social contact survey in Hong Kong in 2015/16 using both paper and online questionnaires. Participants using paper questionnaires reported more contacts and longer contact duration than those using online questionnaires. Participants reported 13 person-hours of contact and 8 contacts per day on average, which decreased over age but increased with household size, years of education and income level. Prolonged and frequent contacts, and contacts at home, school and work were more likely to involve physical contacts. Strong age-assortativity was observed in all age groups. We evaluated the characteristics of social contact patterns relevant to the spread of respiratory infectious diseases in Hong Kong. Our findings could help to improve the design of future social contact surveys, parameterize transmission models of respiratory infectious diseases, and inform intervention strategies based on model outputs.

## Introduction

Many respiratory infectious diseases are transmitted through close person-to-person contacts. Therefore, predicting infection spread and the impact of interventions such as vaccination relies on being able to quantify close contacts among individuals, particularly how different age groups mix. Consequently, large population-based surveys of social contacts targeted at understanding the spread of respiratory infections have been conducted in different populations^1–22^. The most common method to measure social contacts is to ask participants to report the number of contacts they make among different age groups on a given day, the proportion of contacts made in different social settings, the duration of contacts, as well as other characteristics regarded to be important for disease transmission (e.g. age, household size)^1–22^. These social contact data are then used to parameterize mathematical models to capture the transmission patterns of many respiratory infections, such as pertussis^23^, influenza^12, 13, 22, 24–27^, respiratory syncytial virus^28^ and varicella^29–31^. However, these data are only available in a limited number of populations such as selected European countries^1, 2, 4–8, 10, 22^, Japan^9^, Guangdong^11^, Taiwan^14^, Vietnam^15^, Thailand^17^, Peru^18^, Kenya^19^, Zambia^20^, Zimbabwe^21^ and South Africa^20^. Although Hong Kong is a hotspot and hub for emerging infectious diseases due to its very high population density and high connectivity in the worldwide air-transportation network^32^, age-specific social contact data for Hong Kong have not yet been published. A social contact survey embedded with a serological survey was conducted in Hong Kong in 2009^13, 33^, but the age of contacts was only reported for a few age groups, and therefore it is difficult to construct customized age-specific contact matrices from the available data.

Previous social contact surveys have used a variety of methods (Supplementary Table S1). Both paper and online questionnaires have been used: paper questionnaires were used in all large-scale population-wide surveys; online questionnaires were first tested in a pilot survey among trained university students in Belgium in 2003^1^ and further compared with paper questionnaires in a comparison study in Australia in 2008^2^; in the later population-wide social contact surveys, online questionnaires were used in parallel with paper questionnaires in UK^6^ and as the main questionnaire mode for all age groups except the elderly aged over 65 in Japan^9^. Social contact data were mainly collected in two ways: 1) trained interviewers completed the questionnaires by asking participants to retrospectively describe their activities during the previous day^11–15, 18, 20^; 2) otherwise, participants were instructed to prospectively record each contact made during the assigned day as it occurred^4, 10, 16, 19, 21^. It is unclear whether the mode of questionnaire type (paper vs. electronic) or the recording behaviors of participants (prospectively vs. retrospectively filling in the questionnaires) would have any substantial impact on the reported contact data, which would further affect the robustness of many modeling studies^12, 13, 22–31^.

To better parameterize modeling studies of respiratory infections in Hong Kong, we conducted a population-based social contact survey in 2015/16 and compared the contact data with those obtained from other countries and regions. In addition, we investigated the impact of different questionnaire mediums and recording behaviors of participants on the reported contact patterns.

## Results

### Reported contacts and the effect of mode of questionnaire

We conducted a population-based social contact survey in Hong Kong using participant-completed diaries similar to those used in the European POLYMOD study by Mossong *et al*.^4^. We defined 15 age groups and applied quota-sampling by age and gender. Children and adolescents below 18 years old were oversampled because they are considered as the main driver for transmission of many respiratory infectious diseases (Table 1). To facilitate recruitment and encourage participant compliance, a paper-based and an electronic online questionnaire with the same contents were developed based on the sample questionnaire from the POLYMOD study and participants were invited to choose the questionnaire mode that they were more comfortable with (Supplementary Text S1).

**Table 1:** Mean number of reported contacts and mean total contact person hours by participant gender, age, day of the week, household size, education, income level and mode of questionnaire.

	All questionnaires			Paper questionnaires			Online questionnaires		
	Number of participants	Average number of reported contacts (SEM)	Average total contact person hours (SEM)	Number of participants (%)	Average number of reported contacts (SEM)	Average total contact person hours (SEM)	Number of participants (%)	Average number of reported contacts (SEM)	Average total contact person hours (SEM)
**Total**	1,149	6.93 (0.19)	11.46 (0.38)	430 (37%)	9.99 (0.39)	15.00 (0.81)	719 (63%)	5.10 (0.17)	9.34 (0.34)
**Gender**									
Male	557	6.98 (0.28)	11.43 (0.57)	206 (37%)	9.99 (0.57)	15.88 (1.24)	351 (63%)	5.21 (0.25)	8.88 (0.49)
Female	592	6.88 (0.27)	11.48 (0.51)	224 (38%)	9.99 (0.54)	14.28 (1.07)	368 (62%)	4.99 (0.23)	9.78 (0.48)
**Age**									
0–10	156	7.65 (0.56)	15.09 (1.01)	60 (38%)	11.18 (1.01)	18.43 (1.85)	96 (62%)	5.45 (0.55)	13.00 (1.12)
11–20	196	6.28 (0.49)	11.61 (1.00)	39 (20%)	13.10 (1.61)	22.30 (3.25)	157 (80%)	4.59 (0.35)	8.96 (0.83)
21–40	334	6.29 (0.32)	10.61 (0.71)	57 (17%)	10.96 (1.21)	18.00 (3.15)	277 (83%)	5.32 (0.26)	9.09 (0.52)
41–65	388	7.44 (0.33)	11.31 (0.65)	201 (52%)	9.66 (0.52)	14.19 (1.11)	187 (48%)	5.05 (0.32)	8.22 (0.54)
> 65	75	7.31 (0.91)	8.05 (1.06)	73 (97%)	7.47 (0.92)	8.15 (1.09)	2 (3%)	1.50 (0.50)	4.31 (0.31)
**Day of the week**									
Weekday	847	6.98 (0.23)	11.55 (0.45)	339 (40%)	9.89 (0.44)	15.18 (0.91)	508 (60%)	5.03 (0.20)	9.13 (0.40)
Weekend	302	6.78 (0.37)	11.20 (0.73)	91 (30%)	10.35 (0.90)	14.31 (1.81)	211 (70%)	5.25 (0.31)	9.86 (0.67)
**Household size**									
1	51	5.12 (0.65)	6.94 (1.07)	22 (43%)	6.59 (0.97)	5.26 (1.43)	29 (57%)	4.00 (0.82)	8.22 (1.52)
2	178	6.38 (0.56)	9.06 (1.11)	88 (49%)	8.42 (0.99)	10.93 (2.07)	90 (51%)	4.39 (0.44)	7.23 (0.80)
3	307	6.57 (0.33)	10.89 (0.62)	108 (35%)	9.31 (0.64)	14.36 (1.15)	199 (65%)	5.09 (0.34)	9.01 (0.69)
4	380	6.93 (0.33)	11.67 (0.59)	129 (34%)	10.90 (0.73)	16.29 (1.31)	251 (66%)	4.89 (0.26)	9.29 (0.52)
5 or above	233	8.21 (0.46)	14.69 (1.05)	83 (36%)	12.01 (0.93)	20.70 (2.38)	150 (64%)	6.11 (0.40)	11.37 (0.87)
**Education**									
Primary or below	250	7.86 (0.45)	13.77 (0.86)	129 (52%)	10.34 (0.69)	15.26 (1.37)	121 (48%)	5.22 (0.47)	12.19 (1.01)
Secondary	384	6.73 (0.34)	10.95 (0.60)	186 (48%)	9.40 (0.57)	14.13 (1.00)	198 (52%)	4.22 (0.29)	7.97 (0.62)
Post-secondary	468	6.72 (0.29)	10.78 (0.63)	91 (19%)	11.40 (1.00)	17.65 (2.57)	377 (81%)	5.59 (0.23)	9.12 (0.44)
**Income**									
0–9999	187	7.30 (0.53)	11.08 (1.03)	114 (61%)	8.91 (0.78)	12.06 (1.50)	73 (39%)	4.79 (0.53)	9.54 (1.20)
10,000–19,999	223	7.00 (0.45)	11.62 (1.01)	104 (46%)	9.80 (0.81)	16.08 (1.91)	119 (53%)	4.55 (0.34)	7.72 (0.73)
20,000–39,999	225	7.88 (0.44)	12.95 (0.84)	85 (38%)	11.04 (0.82)	16.88 (1.70)	140 (62%)	5.97 (0.42)	10.56 (0.81)
≥ 40,000	186	7.53 (0.46)	12.36 (0.89)	40 (22%)	12.20 (1.40)	17.58 (2.74)	146 (78%)	6.25 (0.39)	10.93 (0.82)
Unknown	328	5.67 (0.33)	10.04 (0.62)	87 (27%)	9.57 (0.88)	14.52 (1.65)	241 (73%)	4.26 (0.28)	8.42 (0.56)

**Figure 1:**
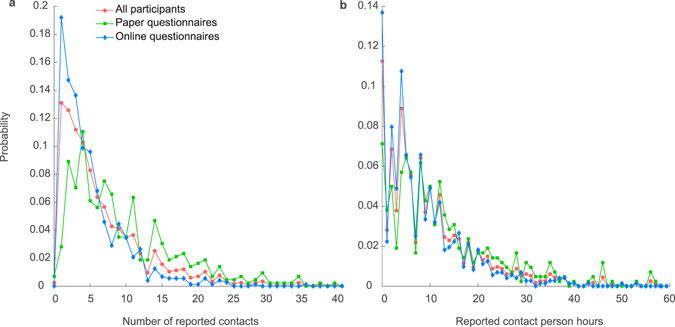
The distribution of the number of reported contacts and total contact duration.

We recorded 7,960 contacts from 557 male respondents and 592 female respondents (Table 1). The mean number of reported daily contacts is 6.93 (95% CI 6.56–7.32), which is smaller than that reported in Japan^9^ and most countries in Europe^4^, but comparable to Germany (7.95 contacts on average)^4^ and Vietnam (7.7 contacts on average)^15^. The distribution of the number of contacts is highly right-skewed with 13 (1.1%) participants reporting more than 30 contacts per day (Fig. 1). We found that participant age, household size, education, income level and mode of questionnaire were significantly associated with the number of reported contacts in the Kruskal-Wallis tests. Participants using paper questionnaires reported on average 9.99 (95% CI 9.24–10.8) contacts per day, which was substantially higher than participants using electronic online questionnaires who on average reported only 5.10 (95% CI 4.78–5.45) contacts per day. The difference is statistically significant (Mann-Whitney U test, p < 0.01). The average number and duration of contacts were 8.14 (95% CI: 7.11–9.31) and 12.77 hours (95% CI: 11.19–14.35) after the mode of questionnaire was considered in the propensity score analysis. The distribution of total contact duration by participant characteristics showed similar pattern as the number of reported contacts (Table 1).

However, the mode of questionnaire was strongly associated with participant age, education and income level in our sample, and therefore its effect on the number of reported contacts and contact duration may be confounded (Table 2). In the mediation analysis, the number of contacts reported in paper questionnaires was found to be significantly higher than online questionnaires after other participant characteristics were considered (Table 2 and Supplementary Table S2). The relative number of reported contacts was 2.32 (95% CI 2.26–2.38).

**Table 2:** The relative number of reported contacts and relative total contact person hours per participant per day by different characteristics of the survey participants.

	Use of paper questionnaire	Relative number of reported contacts		Relative total contact person hours	
	Odds ratio	No propensity score matching	With propensity score matching	No propensity score matching	With propensity score matching
**Gender**					
Male	REF	REF	REF	REF	REF
Female	1.041 (0.773–1.402)	1.000 (0.978–1.023)	1.061 (1.034–1.088)*	1.040 (1.013–1.069)*	1.064 (1.032–1.097)*
**Age**					
0–10	REF	REF	REF	REF	REF
11–20	1.103 (0.608–1.994)	0.926 (0.888–0.967)*	0.953 (0.911–0.996)*	0.839 (0.797–0.882)*	0.847 (0.804–0.893)*
21–40	1.704 (0.861–3.399)	0.847 (0.805–0.891)*	0.746 (0.704–0.790)*	0.726 (0.683–0.772)*	0.640 (0.596–0.687)*
41–65	6.132 (3.327–11.56)*	0.812 (0.776–0.849)*	0.751 (0.717–0.786)*	0.648 (0.613–0.685)*	0.608 (0.575–0.644)*
>65	246.2 (63.06–1657)*	0.608 (0.566–0.653)*	0.562 (0.523–0.604)*	0.396 (0.364–0.432)*	0.379 (0.348–0.413)*
**Day of the week**					
Weekday	REF	REF	REF	REF	REF
Weekend	0.582 (0.411–0.818)*	1.101 (1.074–1.130)*	1.060 (1.029–1.092)*	1.088 (1.055–1.123)*	1.033 (0.997–1.071)
**Household size**					
1	REF				
2	1.556 (0.646–3.912)	1.068 (1.059–1.078)*	1.064 (1.054–1.075)*	1.088 (1.055–1.123)*	1.105 (1.902–1.119)*
3	1.094 (0.472–2.660)	for household size	for household size	for household size	for household size
4	1.390 (0.604–3.368)	increased by one	increased by one	increased by one	increased by one
5 or above	1.569 (0.664–3.895)				
**Education**					
Primary or below	REF	REF	REF	REF	REF
Secondary	0.318 (0.183–0.514)*	0.992 (0.954–1.032)	1.052 (1.009–1.096)*	1.045 (0.997–1.096)	1.082 (1.029–1.139)*
Post-secondary	0.102 (0.056–0.180)*	1.231 (1.179–1.286)*	1.388 (1.322–1.458)*	1.139 (1.081–1.200)*	1.310 (1.234–1.391)*
**Income**					
0–9999	REF	REF	REF	REF	REF
10,000–19,999	0.944 (0.579–1.538)	0.987 (0.949–1.027)	0.963 (0.924–1.004)	1.052 (1.003–1.103)*	1.033 (0.983–1.086)
20,000–39,999	0.585 (0.356–0.959)*	1.184 (1.140–1.230)*	1.142 (1.096–1.190)*	1.248 (1.191–1.306)*	1.179 (1.112–1.238)*
≥40,000	0.256 (0.149–0.435)*	1.277 (1.225–1.330)*	1.284 (1.226–1.344)*	1.313 (1.250–1.379)*	1.284 (1.216–1.355)*
Unknown	0.235 (0.145–0.375)*	0.977 (0.943–1.012)	0.940 (0.904–0.977)*	1.019 (0.977–1.062)	0.936 (0.894–0.979)*
**Mode of survey**					
Online	—	REF	REF	REF	REF
Paper	—	2.321 (2.260–2.383)*	2.324 (2.279–2.406)*	1.882 (1.824–1.943)*	1.893 (1.833–1.955)*

The number of reported contacts and total contact duration decreased over age but increased with household size, education and income level (Table 2). The decline in contact duration over age was more apparent than the number of reported contacts. In contrast to the European and Japan data, we found the difference in weekday and weekend contacts were age-dependent (Supplementary Table S5)^4, 9^. The overdispersion parameter of the negative binomial regression model was significantly larger than zero, suggesting that the model is more appropriate than a Poisson regression model. Despite the small number of prospective participants identified (see details in Methods), there was a statistically significant difference in the number of reported contacts between prospectively and retrospectively filled-in online questionnaires (Supplementary Table S4).

### Nature, duration, location and frequency of contacts

**Figure 2:**
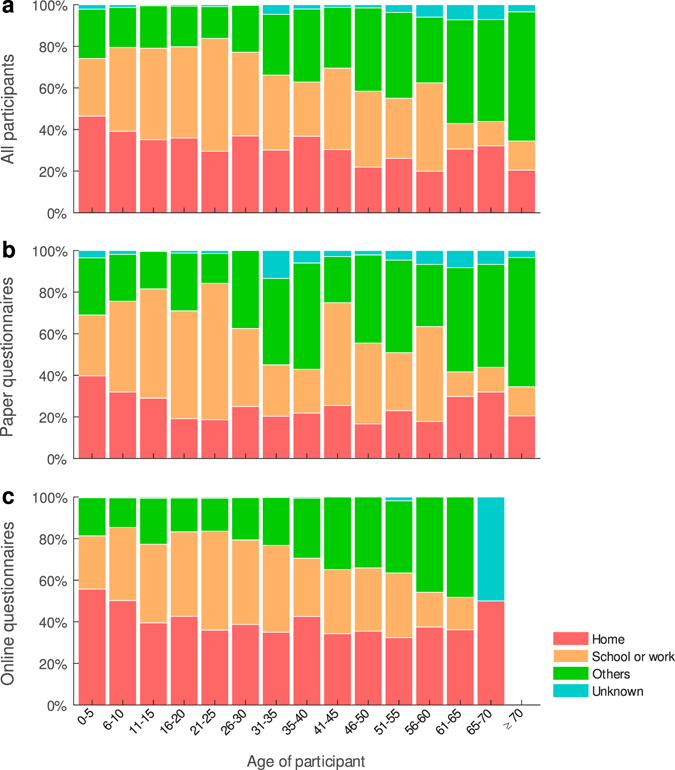
The relation between participants and their contacts. Participants could report the relationship with their contacts in one of the following categories: household members, classmates or schoolmates, workmates, others and unknown. (**a**) All participants. (**b**) Participants using paper questionnaires. (**c**) Participants using online questionnaires.

Our findings show that participants made most of their reported contacts with their home, school and work contacts (Fig. 2). Even though participants using online questionnaires reported fewer contacts than those using paper questionnaires, we found that the online participants reported a higher percentage of contacts with household members. This suggests that contacts with household members were less likely to be left out.

**Figure 3:**
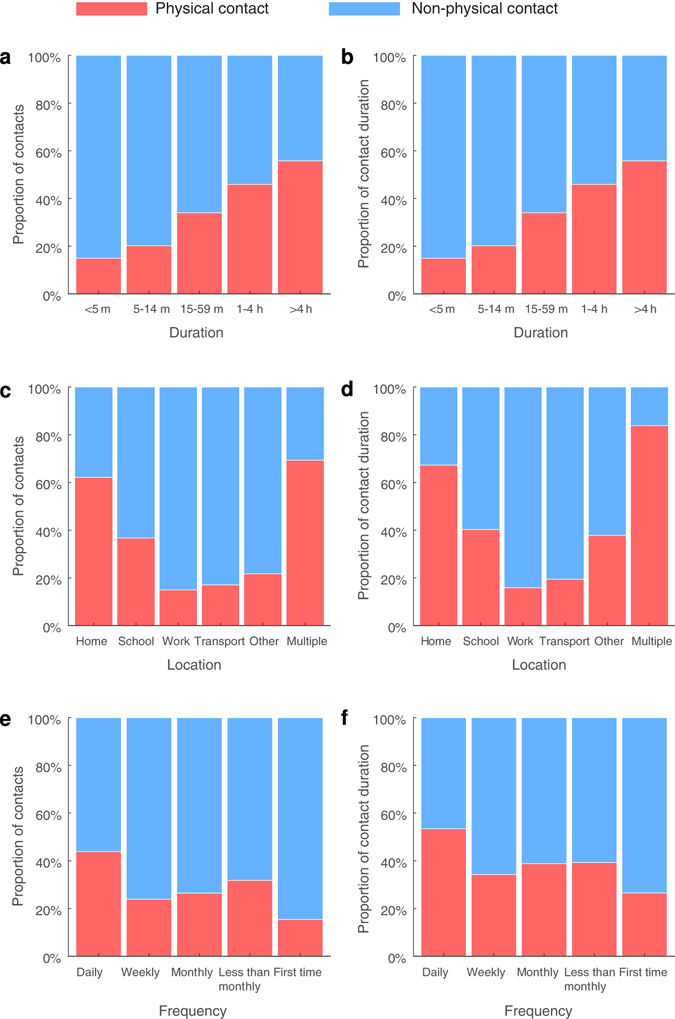
The proportion of contacts and contact duration involving physical contacts. The proportion of contacts and contact duration (in person hours) that were physical or non-physical by (**a**,**b**) duration, (**c**,**d**) location, and (**e**,**f**) frequency of contacts.

We measured the intensity of contacts in several ways, including duration, location, frequency and whether they involved physical contact. Contacts at home, contacts of long duration and contacts of daily frequency were more likely to involve physical contact (Fig. 3). Home contacts were most likely (60%) to involve physical contact, followed by contacts at school and in the workplace. The majority of contacts in multiple locations involved physical contact, likely because most of them involved a contact at home or at school. Similarly, about 50% of contacts longer than one hour involved physical contact. More than 40% of daily contacts involved physical contact, but in contrast only about 15% of contacts with individuals met for the first time involved physical contact.

**Figure 4:**
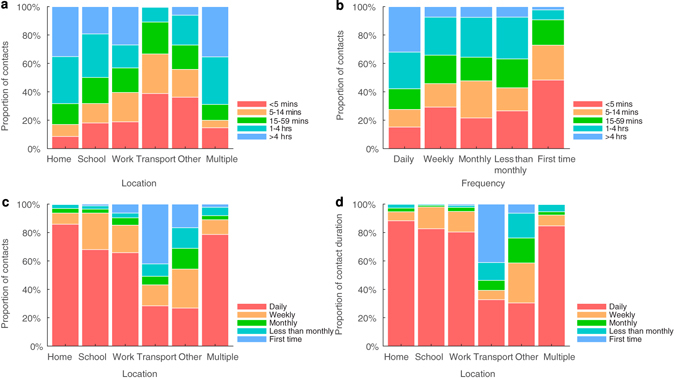
The correlation between contact duration, location and frequency. The correlation between (**a**) duration and location, (**b**) duration and frequency, and (**c**,**d**) location and frequency of contacts.

Contact duration, location and frequency appeared to be associated with each other (Fig. 4). Nearly 70% of contacts at home were longer than one hour, followed by contacts at school (~50%) and in the workplace (~40%). About 60% of daily contacts were for more than one hour while 70% of contacts with individuals met for the first time were shorter than 15 mins. More than 80% of contacts at home were daily contacts, followed by contacts at school (>60%) and in the workplace (~60%).

### Age-related social mixing pattern

**Figure 5:**
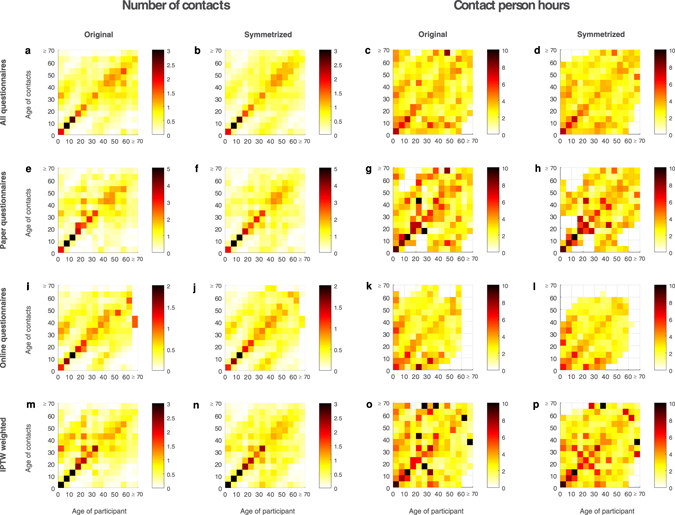
Contact matrix of reported contacts consisting of the average number of contacts and mean contact duration per day per participant. (**a**–**d**) All reported contacts; (**e**–**h**) Contacts reported in paper questionnaires; (**i**–**l**) Contacts reported in online questionnaires; (**m**–**p**) Reported contacts weighted by inverse probability of treatment weighting (IPTW) using propensity scores for mode of questionnaire. The original contact matrices {*c*_*ij*_} show the average number of contacts or mean contact person hours in age group *i* reported by participants from age group *j*; the symmetrized contact matrices are calculated using $\{\widehat{{c}_{ij}}\}=\frac{{c}_{ij}+{c}_{ji}}{2}$.

**Figure 6:**
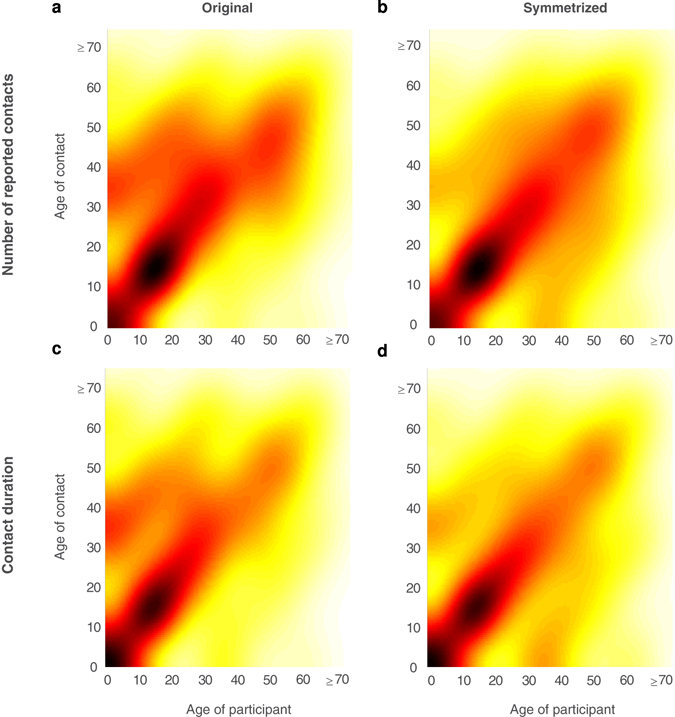
Smoothed contact matrix of all reported contacts and total contact durations. The smoothed contact matrices are constructed based on all contact data using kernel density estimation with a Gaussian kernel: (**a**) original number of reported contacts, (**b**) symmetrized number of reported contacts, (**c**) original contact person hours and (**d**) symmetrized contact person hours. The original contact matrices {*c*_*ij*_} show the average number of contacts or mean contact person hours in age group *i* reported by participants from age group *j*; the symmetrized contact matrices are calculated using $\{\widehat{{c}_{ij}}\}=\frac{{c}_{ij}+{c}_{ji}}{2}$. Inverse probability of treatment weighting (IPTW) was applied with the propensity scores for mode of survey. The bandwidth was optimized by default to estimate normal densities in MATLAB 9.0 and boundary bias were corrected with simple reflection of data.

Figures 5 and 6 (Supplementary Table S6a–d) show the average contact number and duration reported per participant per day with individuals from different age groups. The most apparent feature of the contact matrix is the highest intensity diagonal, demonstrating the age-assortative mixing pattern, i.e. individuals tend to have more contacts with other individuals of similar age. Age assortativity is most pronounced in school children aged 5–20 years old and least apparent in the elderly aged above 65 years old. Two parallel secondary diagonals starting at 30–35 years for both participants and contacts are offset to the central diagonal, showing increased contact intensity between participants contacting with their parents or children across all age groups. The secondary diagonal was more obvious starting from the 30–35 years old contacts. For working-age adults, there is a wide contact intensity plateau at 25–60 years for both participants and contacts involving contacts at the workplace. We explored the potential effects of different social contact data in the estimation of influenza infection attack rates in an age-stratified influenza transmission model we used for the influenza pandemic in 2009^26^, and found that the difference did not generate substantial differences in the model estimates (Supplementary Information).

## Discussion

Using similar methodology to a large European contact survey^4^, we examined social contacts and quantified the contact mixing pattern in Hong Kong. Overall, we recorded a mean of 8.1 contacts per participant per day, which was substantially lower than the 13.4 contacts in the European data and 18.0 contacts reported in the previous Hong Kong contact survey^13^ (Supplementary Table S1). Participants using paper questionnaires reported a significantly greater number of contacts and longer contact duration than those using online questionnaires, and the choice of questionnaire was strongly associated with age, education and income level (Table 1). Similar to the European data, we found significant overdispersion in contact number and total contact duration, and pronounced assortativity in the age-specific contact matrix.

The distributions of both the number of reported contacts and the reported contact duration were highly right-skewed, but the latter distribution had a heavier right tail (Fig. 1). The differences in the two distributions were more obvious in previous contact surveys which allowed participants to include “group contacts”^6, 11^. However, for respiratory infections, there is a lack of empirical data to validate the relationship between the likelihood that a contact between susceptible and infectious individuals transmits infection, and the physicality and duration of the contact^3^.

We found participants with more years of education and higher income levels were more likely to choose online questionnaires, and to report more contacts, after adjusting for questionnaire mode in the propensity score analysis. The finding was consistent with time use data from census statistics^34^, showing a tendency for individuals with more years of education and higher income levels to spend more time on social and leisure activities, while the individuals with less education and lower income levels spent more time on household commitments. In contrast to the European and Japanese data, we found the difference in weekday and weekend contacts was age-dependent (Supplementary Table S5). Children and adolescents under 18 reported more school contacts during weekends than weekdays, which might be due to the Hong Kong schooling system in which students participate in many extra-curricular activities with their schoolmates during weekends, especially on Saturdays. For adults aged 18 to 50, there was no significant reduction in work contacts, and the slight (non-significant) reduction was partly compensated by the increase in contacts with other individuals outside home, school or work during weekends. For older adults aged over 50, the pattern was consistent with other contact surveys and a decrease in all contact types was observed. Given the relatively small number of reported contacts in our study, the lower number of weekday reported contacts might partly stem from recall bias because short-lived contacts and work contacts were more likely to be reported as forgotten^3^.

The contact intensity was highest among school-aged children aged below 20 and decreased over age. Participants were more likely to contact their family members, schoolmates and workmates. Similarly, prolonged and frequent contacts, and contacts at home, school and work were more likely to involve physical contacts. The strongest age-assortativity was found among school-aged children and adolescents (Figs 5 and 6). We also found another strong area with higher contact intensity in adults aged 41 to 65 years old, which was also observed in Vietnam^15^ but absent in Japanese and European data^4, 9^. However, the contact intensity was reduced in this group after weighting was applied with propensity scores for modes of questionnaire, suggesting this might probably result from the higher use of paper questionnaires among this age group.

The comparison of data obtained from paper and online questionnaires was not part of our original study design. This post-hoc analysis was done because of the apparent difference observed in the number of reported contacts between the two modes. Nonetheless, our study is one of the few contact surveys to investigate both the determinants and outcomes of using paper vs online questionnaires in all age groups^1, 2, 6^. In our study, the choice of online questionnaire was significantly associated with age, education and income level, which was consistent with findings from a study about mixed-mode administration of questionnaires^35^. In contrast to the previous contact surveys^1, 2, 6^, participants using paper questionnaires in our study reported higher number of contacts and longer contact duration than online questionnaires, even after participant characteristics were considered. The only difference between paper and online questionnaires was the format of the contact diary: in the paper questionnaire, we provided 100 empty contact entries in a booklet; in the online questionnaire, the contact diary was a dynamic table where participants could add one entry whenever they clicked the “add contact” button.

It is unclear whether the format of the online questionnaire had discouraged participants from filling in their contacts. Limited literature has been published that directly compares paper and online contact surveys. In surveys conducted by both Beutels *et al*. and McCaw *et al*., participants were instructed to use both paper and online questionnaires simultaneously^1^ or alternatively in two consecutive weeks^2^. McCaw *et al*. conducted the comparison of questionnaire modes in a small social contact survey of 65 adult participants, and found that ascertainment using paper questionnaires was superior to the online questionnaires delivered using PDAs^2^. It is difficult to compare paper and online questionnaires in the two large population-wide contact surveys in UK and Japan: in the Japan study^9^, only those aged over 65 who did not live with younger household members used paper questionnaires; in the UK study^6^, the recruitment methods were different for the two modes, with paper questionnaires distributed to randomly selected households and online questionnaires available to anyone who was interested to participate. More investigation of questionnaire design and administration should be considered in the future contact survey studies, given with the rapidly growing use of online tools for data collection.

We analyzed the effect of prospective and retrospective completion by assessing the actual time that participants filling in each contact entry. We found that more than 95% of participants were likely to have completed the questionnaires retrospectively, even though we encouraged them to do it prospectively. Despite the limited number of prospective participants, we found that more contacts were reported in prospective participants, and the effect was statistically significant among online questionnaires (Supplementary Table S4). The overall low average number of reported contacts in our survey might reflect the recall bias introduced by the retrospective behavior of our participants. While the analyses we were able to do was limited by the small number of prospectively filled questionnaires, more comparisons between prospective and retrospective surveys could be conducted if future contact surveys incorporate information about recording time. With the increasing use of smart phones and wearables, future social contact surveys might also consider using these devices to send out several reminders during the assigned days, or upon changes in the location of participants such as home to school or work to leisure, hence minimizing reliance on participants’ memory to record contact events retrospectively.

Heterogeneities among the various methods used in social contact surveys make it difficult to directly compare contact data obtained from different populations (Supplementary Table S1). Among the surveys conducted in Asia, questionnaires were completed retrospectively by participants in Japan, by interviewers in the previous Hong Kong survey and other regions including Vietnam, Guangdong and Taiwan. The previous Hong Kong study and the Guangdong study asked participants to consider “group contacts” which might have increased the number of reported contacts^11, 13^. Unlike other social contact surveys, participants of the Japan study were recruited from the participant pool maintained routinely by a survey company, and online questionnaires were distributed to most of the participants who were already familiar with different designs of online questionnaires^9^. The contact matrix was not available in the Taiwan study^14^ and cannot be constructed in customized age groups from the previous Hong Kong study^12, 13^. More comparison between different social contact surveys could be done if more detailed documentation of contact data were available online.

In conclusion, we evaluated the characteristics of social contacts and mixing patterns relevant to the spread of respiratory infectious diseases in Hong Kong. Our data provide important information to improve the parameterization of mathematical models for infectious disease transmissions in Hong Kong, especially respiratory infections through close contacts such as varicella, RSV and influenza. Our findings could help to improve the design of future social contact surveys, and inform intervention strategies based on the outputs of modelling studies.

## Methods

### Survey methods

Participants were recruited by random digit dialing of all fixed land-line based residential telephone lines. Each telephone number was dialed for a maximum of 5 times until there was a response. Upon a successful telephone connection, we delivered a brief introduction of the study to the respondent. The respondent was then asked about his/her household composition. One eligible household member was chosen randomly from the pool of people who would fulfil age and sex quotas, and invited to participate in our study. Recruitment continued until we met our predefined target sizes by age and sex.

Three types of questionnaires were provided for participants of different ages: parental-proxy child questionnaires for 0 to 10 year olds, self-reported adolescent questionnaires for 11 to 17 year olds, and self-reported adult questionnaires for 18 year olds or above. We adopted the same contact definition as the POLYMOD study^4^: a contact was defined as either skin-to-skin touch such as a handshake (a physical contact) or a face-to-face conversation with three or more words in the physical presence of both the participant and the contact within two meters. Participants were instructed to make one entry for each person contacted between 5 am of the assigned day and 5 am of the day after, regardless of the number of contacts with that person. Information was obtained about the age and gender of each contact, the duration and location of the contact, whether physical contact was involved, and how often the participant met with the contact. Participants were encouraged through the instructions to fill each contact in the questionnaire prospectively (as soon as they ended each contact) rather than retrospectively (at the end of the day). In common with other diary-based questionnaires, we had no way of ensuring that they actually did this. However, we had information about the actual time that participants made their entries, either by saving the computer record of time (in the online questionnaire system), or by asking participants to manually record the time at which they filled in each contact (in the paper questionnaire). In both cases, we classified participants as having filled in the questionnaires retrospectively if there was less than one-hour difference between the time of the first and last contact in the questionnaire, and prospectively otherwise. Participants with more than 24-hours difference in the time elapsed and participants who had recorded only a single contact were not included in the comparison of prospective and retrospective questionnaires.

### Statistical analysis

We assessed the effect of participant characteristics (i.e. gender, age, day of the week, household size, education, income level and mode of questionnaire) on the number of reported contacts using non-parametric Kruskal-Wallis test. We applied mediation analysis to show the mode of questionnaire was a mediator in the causal relationship between participant characteristics and the number of reported contacts (Supplementary Table S2)^36^. In the mediation analysis we followed the Step 1–3 described by David A. Kenny^36^ as follows:

1. We showed that the number of reported contacts was associated with participant gender, age, day of the week, household size, education and income level in a negative binomial multivariate regression model.
2. We showed that the choice of questionnaire mode was associated with participant gender, age, day of the week, household size, education and income level in a logistic regression model.
3. We showed that the number of reported contacts was associated with choice of questionnaire mode, age, gender, day of the week, household size, education and income level.

Then we applied propensity score analysis to reduce the potential effects due to selected mode of questionnaire medium^37^. The propensity of choosing a paper questionnaire was estimated for each participant in the logistic regression model shown in the above Step 2 (Supplementary Figure S1). We created a synthetic sample by matching the resulting propensity scores using the “MatchIt” package in R 3.3.3^38^. In the matched sample, we compared the subjects in paper and online questionnaire group by computing the standardized difference of their demographic variables (Supplementary Table S3).

With both the original and synthetic sample, we used a weighted multivariable negative binomial regression model to assess the effects of covariates (i.e. gender, age, day of the week, household size, education, income level and mode of questionnaire) on the number of reported contacts and the total contact duration (in person hours). The duration of each contact event was reported in one of four categories, so we assigned contact duration as the midpoint of the corresponding category right censored at 4 hours. Then we calculated the total contact duration of each participant. The sampling weights were calculated based on the age distribution from the Hong Kong census in 2015. The distribution of household sizes was not considered because we were not able to obtain census statistics about household sizes stratified by age. In a sensitivity analysis, we compared the negative binomial regression model with a weighted multivariable Poisson regression model, and found that the negative binomial regression model had a lower AIC. Smoothing of the age-specific contact matrix was performed using bivariate kernel density estimation with a Gaussian kernel. Inverse probability of treatment weighting (IPTW) was used in the smoothing, with the propensity scores for the mode of questionnaire. The bandwidth was optimized for estimating normal densities in MATLAB 9.0. Boundary bias were corrected using simple reflection of contact data^39^.

### Ethical approval

The study was approved by Institutional Review Board of The University of Hong Kong/Hospital Authority Hong Kong West Cluster (HKU/HA HKW IRB). The reference number is UW 14–537. All methods were performed in accordance with relevant guidelines and regulations. Informed consent was obtained from all participants and/or their legal guardians.

### Data availability

The data collected as part of this survey will be made available to the scientific community via the zenodo data repository as part of a social contact data collection initiative www.socialcontactdata.org thanks to ERC grant TransMID (grant agreement 682540) awarded to Niel Hens (Hasselt University and University of Antwerp).

## Electronic supplementary material

Supplementary Information
