# Supplementary material for: Social contact patterns relevant to the spread of respiratory infectious diseases in Hong Kong
Source: Sci Rep. 2017 Aug 11;7:7974. doi: 10.1038/s41598-017-08241-1 (PMC5554254; doi:10.1038/s41598-017-08241-1)
Supplement: Supplementary file 1 — Supplementary Information [file 41598_2017_8241_MOESM1_ESM.pdf]

Social contact patterns relevant to the spread of respiratory infectious diseases in Hong Kong

Kathy Leung[1,a], Mark Jit[1,2,3,a], Eric H. Y. Lau[1], Joseph T. Wu[1*]

[1] WHO Collaborating Centre for Infectious Disease Epidemiology and Control, School of Public Health, Li Ka Shing Faculty of Medicine, The University of Hong Kong, Hong Kong SAR, People's Republic of China

[2] Department of Infectious Disease Epidemiology, London School of Hygiene and Tropical Medicine, London, United Kingdom

[3] Modelling and Economics Unit, Public Health England, London, United Kingdom

[a] Contributed equally

*Corresponding author:

Dr. Joseph T. Wu
WHO Collaborating Centre for Infectious Disease Epidemiology and Control
School of Public Health
Li Ka Shing Faculty of Medicine
The University of Hong Kong
Tel: +852 3917 6709
Email: joewu@hku.hk

Supplementary Information

The impact of different social contact data on the estimation of influenza infection attack rates

We explored whether the difference in social contact patterns between Hong Kong and European countries would result in significant difference in the estimates of age-specific influenza infection attack rates (IARs). In our previous study of the 2009 influenza pandemic [1], we estimated the age-specific infection attack rates in Hong Kong from a Susceptible-Exposed-Infectious-Removed model constructed based on the European social contact data [2]. We showed that the estimates of age-specific infection attack rates were similar when different contact matrices from the POLYMOD study were used. To investigate whether the IAR estimates are sensitive to the difference between Hong Kong contact data and European contact data, we replicated the study with Hong Kong contact data from this study.

Supplementary Figure S2 compares the IAR estimates across all the age groups when different contact matrices were used. The IAR estimates were largely consistent, except that the IAR of 13-19 years old was slightly higher when Hong Kong contact data were used. In this case study, we showed that the structure of contact matrices had little impact on the age-specific IAR estimates. Given any contact matrix, the parameter "age-specific susceptibility" would be adjusted in the Bayesian inference algorithm accordingly so that similar transmission dynamics could be generated to match the observed transmission patterns. The high age-assortative within-group contact intensity along the diagonal of contact matrices is the most important finding in all the social contact surveys, and plays a major part in the estimation of age-specific infection attack rates and susceptibility.

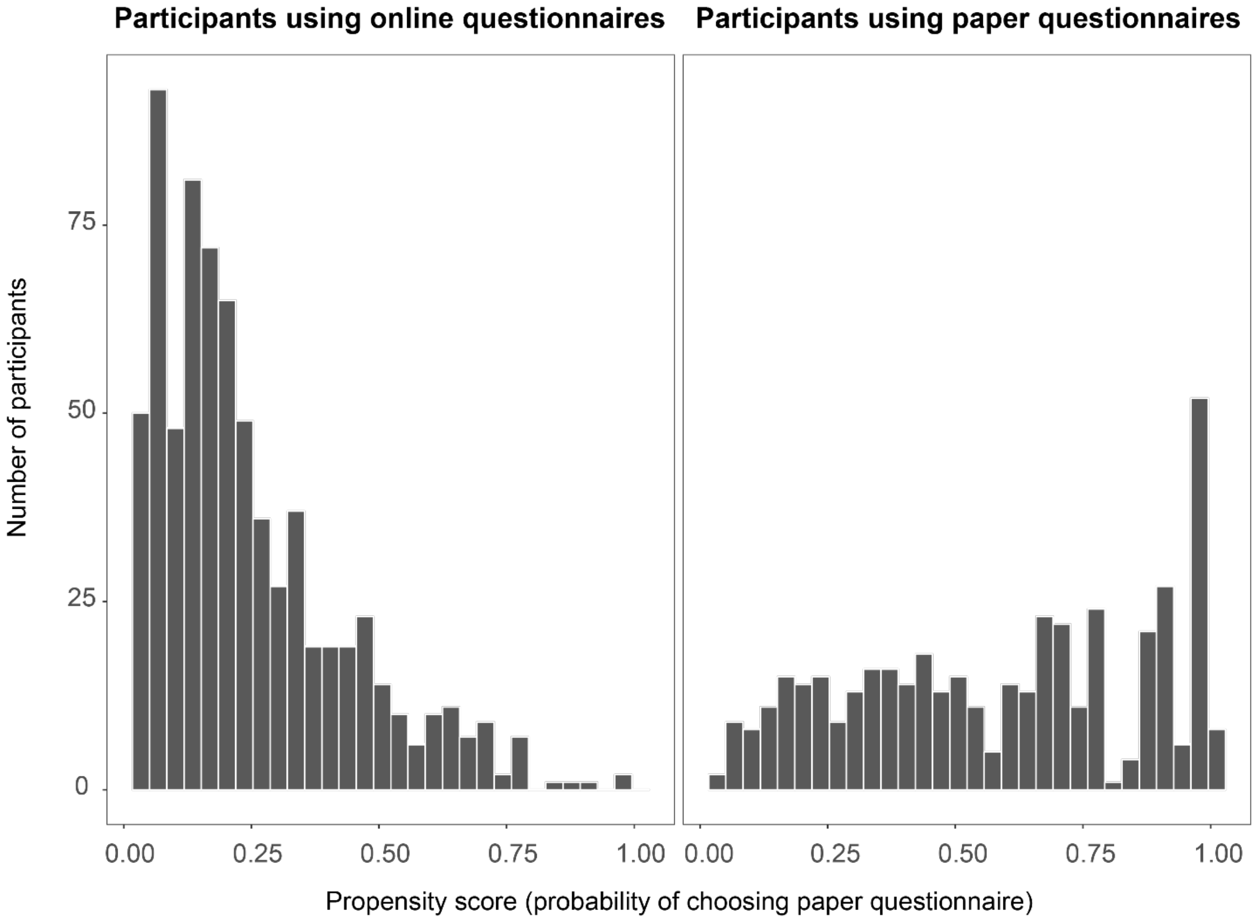

Supplementary Figure S1. The distribution of estimated propensity scores for mode of questionnaire by the actual type of questionnaire chosen by each participant

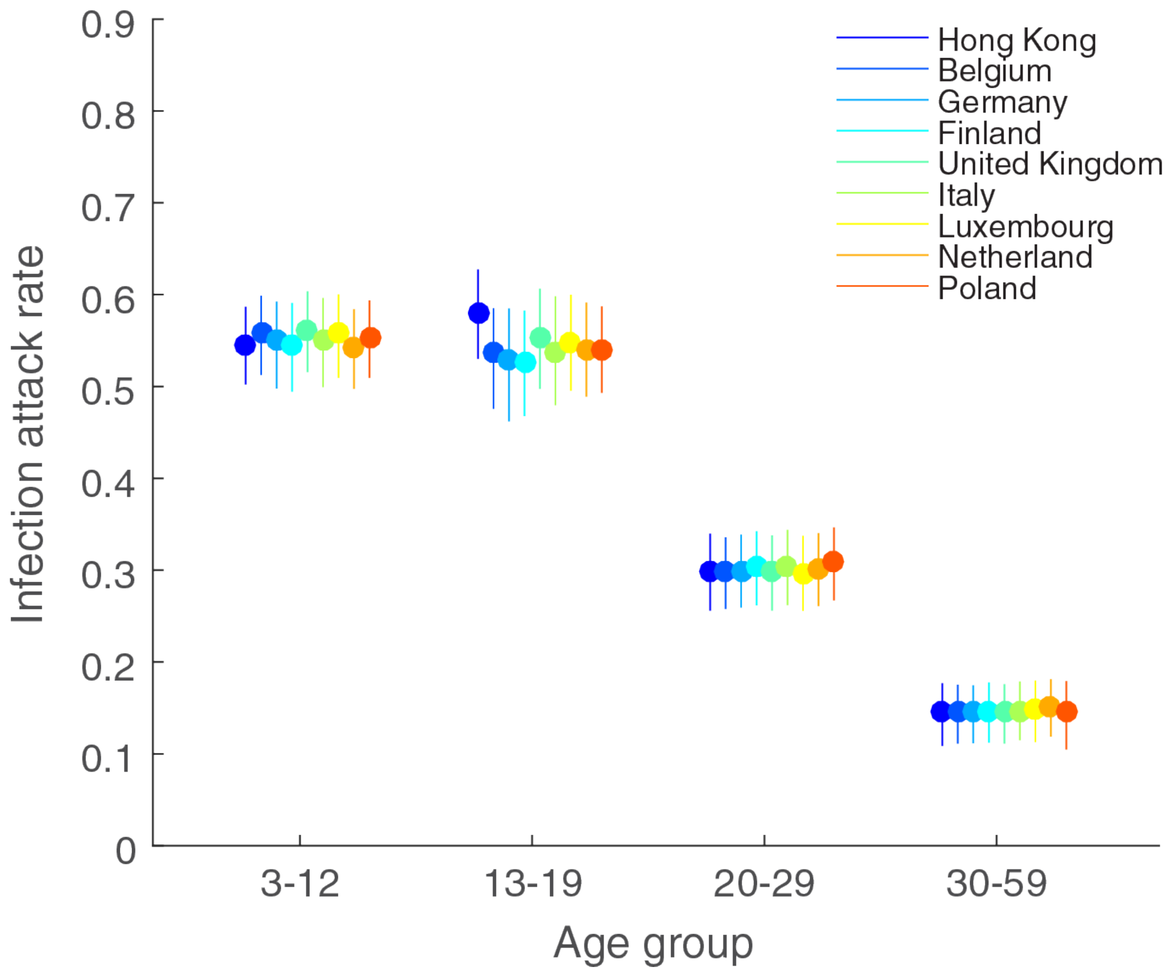

Supplementary Figure S2. Estimates of age-specific infection attack rates of the 2009 influenza pandemic in Hong Kong when different contact matrices were assumed.

The Hong Kong contact matrix was constructed with contact data in this manuscript after questionnaire mode was considered in the propensity score analysis. The contact matrices of eight European countries were obtained from Mossong et al [2].

Study	Country or region	Year of study	Mode of data collection	Mode of questionnaire	Prospective or retrospective	Include group contacts	Mean number of reported contacts
Beutals	Belgium	2003	Self-report	Online	Both	No	23.5 on weekdays and 19.5 during weekends among trained university students
Mossong	Eight European countries	2006	Self-report	Paper	Not specified	No	7.95-19.77
Horby	Vietnam	2007	Interview	Paper	Retrospective	No	7.7
McCaw	Australia	2008	Self-report	Paper and PDA	Prospective	No	20.3-27.5 among 65 adults using both modes
Danon	UK	2009	Self-report	Paper and online	Not specified	Yes	7.97 (without group contacts)
Kucharski and Kwok	Hong Kong	2009	Interview	Paper	Retrospective	Yes	18.0-18.6
Read	Guangdong	2010	Interview	Paper	Retrospective	Yes	18.56
Fu	Taiwan	2010	Interview	Paper	Retrospective	Not clear	12.54
Johnstone-Robertson	South Africa	2010	Self-report	Paper	Prospective (encouraged)	No	15.8
Dodd	Zambia and South Africa	2010-2011	Interview	Paper	Retrospective	Both	5.7 (adult participants only, without group contacts)
Kifle	Belgium	2010-2011	Self-report	Paper	Prospective	No	13.5
Ibuka	Japan	2011	Self-report	Paper and online for elderly individuals over 65 years who don't live with younger adults; online for other age groups and elderly aged over 65 living with younger adults	Retrospective	No	15.3
Grijalva	Peru	2011	Interview	Paper	Retrospective	No	12
Kiti	Kenya	2011	Self-report	Paper	Prospective (encouraged)	No	17.7
Beraud	France	2012	Self-report	Paper	Not specified	Both	19.1 (without group contacts)

Melegaro	Zimbabwe	2013	Self-report	Paper	Prospective (encouraged)	No	10.8
Ajelli	Russia	2016	Self-report	Paper	Not specified	No	12.2

Supplementary Table S1. Main features of the previous social contact surveys

We summarized previous population-wide social contact surveys conducted with similar diary design as the POLYMOD study by searching the titles and abstracts of journal articles that have cited Mossong et al [2]. In addition, we also searched the reference list of each article we found to identify additional eligible studies to be included in Table S1.

	**Step 1 (relative number of reported contacts)**	**Step 2 (OR of choosing paper questionnaire)**	**Step 3 (relative number of reported contacts)**
**Gender**			
Male	REF	REF	REF
Female	1.008 (0.915-1.111)	1.036 (0.770-1.393)	1.000 (0.978-1.023)
**Age**			
0-10	REF	REF	REF
11-20	0.942 (0.766-1.161)	1.091 (0.602-1.969)	0.926 (0.888-0.967) *
21-40	0.895 (0.714-1.123)	1.711 (0.867-3.406)	0.847 (0.805-0.891) *
41-65	1.092 (0.892-1.337)	6.219 (3.385-11.69) *	0.812 (0.776-0.849) *
>65	1.190 (0.908-1.565)	256.5 (65.92-1723) *	0.608 (0.566-0.653) *
**Day of the week**			
Weekday	REF	REF	REF
Weekend	0.996 (0.892-1.114)	0.587 (0.415-0.824) *	1.101 (1.074-1.130) *
**Household size**	1.112 (1.061-1.165) *	1.074 (0.927-1.246)	1.068 (1.059-1.078) *
**Education**			
Primary or below	REF	REF	REF
Secondary	0.870 (0.736-1.026)	0.318 (0.183-0.540) *	0.992 (0.954-1.032)
Post-secondary	0.887 (0.741-1.061)	0.102 (0.057-0.181) *	1.231 (1.179-1.286) *
**Income**			
0-9999	REF	REF	REF
10,000-19,999	1.023 (0.867-1.208)	0.937 (0.576-1.522)	0.987 (0.949-1.027)
20,000-39,999	1.130 (0.958-1.331)	0.577 (0.352-0.944) *	1.184 (1.140-1.230) *
⩾40,000	1.052 (0.883-1.254)	0.260 (0.151-0.441) *	1.277 (1.225-1.330) *
Unknown	0.795 (0.682-0.926) *	0.234 (0.145-0.373) *	0.977 (0.943-1.012)
**Mode of questionnaire**			
Online	-	-	REF
Paper	-	-	2.321 (2.260-2.383) *

Supplementary Table S2. Mediation analysis showing mode of questionnaire is a mediator in the causal relationship between demographic characteristics and the number of reported contacts.

	Standardized difference (%)	
	**Before propensity score matching**	**After propensity score matching**
**Gender**	1.83	2.79
**Age**	16.6	7.31
**Day of the week**	-18.9	-12.0
**Household size**	10.1	4.92
**Education**	16.4	5.30
**Income**	13.2	4.55

Supplementary Table S3. Standardized differences in the demographic covariates used in the propensity score estimation.

	Retrospective participants		Prospective participants		Mann-Whitney U test
	Number of participants	Average number of reported contacts (SD)	Number of participants	Average number of reported contacts (SD)	p-value
**Online questionnaire**					
By the time between first and last contact entry	704	5.00 (4.46)	9	9.33 (3.24)	<0.01*
By the time between first and last access	553	5.04 (4.45)	40	6.85 (6.14)	0.035*
**Paper questionnaire**					
By the time between first and last contact entry	420	9.89 (8.06)	6	13.67 (12.60)	0.59

Supplementary Table S4. Number of reported contacts among prospective and retrospective participants.

		Average number of reported contacts by contact type				
	Number of participants	All	Home	School or work	Others	Unknown
**Paper questionnaire**						
**Age 0-18**						
Weekday	61	11.08	3.70	4.18	3.13	0.07
Weekend	31	13.26	4.42	6.03	2.26	0.58
**Age 19-30**						
Weekday	24	13.08	2.25	8.25	2.42	0.17
Weekend	5	8.40	3.20	2.20	2.80	0.20
**Age 31-50**						
Weekday	74	11.32	2.64	4.66	3.65	0.38
Weekend	20	12.10	1.70	4.30	4.55	1.55
**Age >50**						
Weekday	180	10.13	2.31	2.38	4.89	0.56
Weekend	35	7.06	1.46	2.60	2.49	0.51
**Online questionnaire**						
**Age 0-18**						
Weekday	157	4.58	2.31	1.44	0.82	0.01
Weekend	72	5.42	2.18	2.14	1.04	0.06
**Age 19-30**						
Weekday	122	4.89	1.96	2.23	0.68	0.02
Weekend	42	5.38	1.79	2.24	1.33	0.02
**Age 31-50**						
Weekday	171	5.70	2.21	1.78	1.71	0.01
Weekend	70	5.39	1.84	2.09	1.43	0.03
**Age >50**						
Weekday	58	4.64	1.47	1.31	1.86	0.00
Weekend	27	4.22	1.37	1.07	1.59	0.19

Supplementary Table S5. Average number of reported contacts by age and contact member type on weekdays (Mon-Fri) and during weekends (Sat-Sun)

		Age of contact														
		**0-5**	**6-10**	**11-15**	**16-20**	**21-25**	**26-30**	**31-35**	**36-40**	**41-45**	**46-50**	**51-55**	**56-60**	**61-65**	**66-70**	**>70**
**Age of participant**	**0-5**	1.97	0.46	0.13	0.08	0.46	0.70	1.15	0.68	0.49	0.21	0.27	0.32	0.33	0.10	0.04
	**6-10**	0.35	2.99	0.63	0.18	0.22	0.44	0.71	0.85	0.79	0.39	0.14	0.08	0.25	0.14	0.17
	**11-15**	0.04	0.24	2.70	0.33	0.28	0.17	0.26	0.54	0.63	0.47	0.31	0.07	0.10	0.04	0.10
	**16-20**	0.03	0.08	0.38	1.55	0.73	0.23	0.31	0.24	0.36	0.50	0.40	0.19	0.07	0.01	0.01
	**21-25**	0.05	0.06	0.13	0.76	1.40	0.67	0.70	0.43	0.61	0.48	0.62	0.39	0.35	0.02	0.00
	**26-30**	0.23	0.04	0.01	0.23	0.50	0.93	0.83	0.47	0.40	0.33	0.26	0.33	0.30	0.04	0.07
	**31-35**	0.36	0.39	0.22	0.23	0.61	0.78	1.33	0.52	0.69	0.41	0.41	0.32	0.60	0.11	0.17
	**36-40**	0.46	0.49	0.28	0.13	0.31	0.49	0.64	0.90	0.94	0.29	0.28	0.14	0.31	0.08	0.13
	**41-45**	0.17	0.31	0.58	0.19	0.35	0.56	0.78	0.86	1.23	1.09	0.60	0.26	0.32	0.06	0.12
	**46-50**	0.05	0.22	0.32	0.44	0.53	0.62	0.83	0.92	1.16	1.28	1.01	0.40	0.63	0.02	0.18
	**51-55**	0.10	0.18	0.24	0.35	0.63	0.45	0.89	0.47	0.84	1.08	1.04	0.54	0.36	0.08	0.20
	**56-60**	0.65	0.07	0.10	0.21	0.43	0.59	0.88	0.74	1.15	0.88	1.57	0.96	0.79	0.31	0.40
	**61-65**	0.15	0.04	0.07	0.04	0.27	0.40	0.58	0.58	0.42	0.48	0.67	0.90	0.77	0.48	0.22
	**66-70**	0.37	0.00	0.15	0.07	0.41	0.52	0.67	0.59	0.67	0.30	0.52	0.59	0.96	0.37	0.33
	**>70**	0.06	0.03	0.00	0.37	0.26	0.46	0.60	0.80	0.89	0.40	0.29	0.57	1.03	0.77	1.17

Supplementary Table S6a. Contact matrix of all reported contacts consisting of the mean number of contacts per day per participant (similar as Table S5 of Mossong et al)

		Age of contact														
		**0-5**	**6-10**	**11-15**	**16-20**	**21-25**	**26-30**	**31-35**	**36-40**	**41-45**	**46-50**	**51-55**	**56-60**	**61-65**	**66-70**	**>70**
**Age of participant**	**0-5**	3.17	0.58	0.25	0.14	0.67	0.81	1.67	0.75	0.83	0.25	0.44	0.61	0.56	0.19	0.11
	**6-10**	0.62	5.07	0.83	0.38	0.24	0.62	0.79	0.97	1.21	0.62	0.17	0.03	0.24	0.24	0.38
	**11-15**	0.05	0.71	5.24	0.33	0.48	0.19	0.71	0.95	1.29	0.48	0.67	0.00	0.14	0.19	0.38
	**16-20**	0.00	0.08	0.54	3.46	2.92	0.23	1.23	0.31	1.00	0.77	1.23	0.31	0.00	0.00	0.08
	**21-25**	0.13	0.06	0.25	0.25	2.50	1.06	2.19	1.13	2.06	0.38	1.44	0.88	0.69	0.06	0.00
	**26-30**	0.00	0.00	0.00	0.80	1.00	3.00	1.20	0.20	1.00	0.60	0.40	0.20	0.40	0.40	0.40
	**31-35**	0.24	0.38	0.62	0.19	0.95	1.29	2.81	0.57	1.24	0.38	0.48	0.38	0.95	0.10	0.33
	**36-40**	1.00	0.07	0.14	0.21	0.21	0.36	1.21	1.00	1.86	0.57	0.79	0.36	0.64	0.36	0.57
	**41-45**	0.26	0.61	0.52	0.35	0.52	0.96	1.13	1.30	2.17	1.83	1.17	0.61	0.09	0.04	0.30
	**46-50**	0.10	0.40	0.38	0.45	0.60	0.93	1.23	1.55	2.05	2.08	1.75	0.68	0.98	0.05	0.28
	**51-55**	0.17	0.37	0.39	0.41	0.95	0.63	1.46	0.56	1.32	1.10	1.76	0.85	0.44	0.17	0.37
	**56-60**	0.83	0.10	0.13	0.23	0.50	0.71	1.08	0.94	1.48	1.04	2.10	1.23	0.94	0.42	0.54
	**61-65**	0.17	0.05	0.08	0.03	0.28	0.42	0.62	0.63	0.48	0.50	0.68	0.88	0.80	0.53	0.25
	**66-70**	0.38	0.00	0.15	0.08	0.42	0.54	0.69	0.58	0.65	0.31	0.54	0.62	1.00	0.38	0.35
	**>70**	0.06	0.03	0.00	0.37	0.26	0.46	0.60	0.80	0.89	0.40	0.29	0.57	1.03	0.77	1.17

Supplementary Table S6b. Contact matrix of reported contacts from paper questionnaires consisting of the mean number of contacts per day per participant (similar as Table S5 of Mossong et al)

		**Age of contact**														
		**0-5**	**6-10**	**11-15**	**16-20**	**21-25**	**26-30**	**31-35**	**36-40**	**41-45**	**46-50**	**51-55**	**56-60**	**61-65**	**66-70**	**>70**
**Age of participant**	**0-5**	1.18	0.38	0.05	0.04	0.33	0.64	0.82	0.64	0.27	0.18	0.16	0.13	0.18	0.04	0.00
	**6-10**	0.16	1.58	0.49	0.05	0.21	0.33	0.65	0.77	0.51	0.23	0.12	0.12	0.26	0.07	0.02
	**11-15**	0.04	0.11	1.97	0.33	0.22	0.16	0.12	0.42	0.44	0.47	0.21	0.10	0.08	0.00	0.01
	**16-20**	0.03	0.09	0.36	1.29	0.43	0.23	0.18	0.23	0.28	0.46	0.29	0.17	0.09	0.01	0.00
	**21-25**	0.03	0.06	0.10	0.88	1.15	0.57	0.35	0.26	0.26	0.50	0.43	0.28	0.26	0.01	0.00
	**26-30**	0.25	0.05	0.02	0.18	0.46	0.77	0.80	0.49	0.35	0.31	0.25	0.34	0.29	0.02	0.05
	**31-35**	0.39	0.39	0.11	0.24	0.51	0.64	0.91	0.50	0.54	0.42	0.39	0.30	0.50	0.11	0.12
	**36-40**	0.34	0.58	0.31	0.11	0.33	0.52	0.52	0.88	0.73	0.23	0.17	0.09	0.23	0.02	0.03
	**41-45**	0.13	0.19	0.61	0.13	0.28	0.39	0.63	0.67	0.83	0.78	0.35	0.11	0.43	0.07	0.04
	**46-50**	0.00	0.06	0.28	0.43	0.47	0.36	0.49	0.38	0.40	0.60	0.38	0.17	0.34	0.00	0.11
	**51-55**	0.04	0.02	0.10	0.29	0.35	0.29	0.40	0.40	0.44	1.06	0.44	0.27	0.29	0.00	0.06
	**56-60**	0.20	0.00	0.05	0.15	0.25	0.30	0.40	0.25	0.35	0.50	0.30	0.30	0.45	0.05	0.05
	**61-65**	0.08	0.00	0.00	0.08	0.23	0.31	0.38	0.31	0.15	0.38	0.62	1.00	0.62	0.23	0.08
	**66-70**	0.00	0.00	0.00	0.00	0.00	0.00	0.00	1.00	1.00	0.00	0.00	0.00	0.00	0.00	0.00
	**>70**	0.00	0.00	0.00	0.00	0.00	0.00	0.00	0.00	0.00	0.00	0.00	0.00	0.00	0.00	0.00

Supplementary Table S6c. Contact matrix of reported contacts from online questionnaires consisting of the mean number of contacts per day per participant (similar as Table S5 of Mossong et al)

		Age of contact														
		**0-5**	**6-10**	**11-15**	**16-20**	**21-25**	**26-30**	**31-35**	**36-40**	**41-45**	**46-50**	**51-55**	**56-60**	**61-65**	**66-70**	**>70**
**Age of participant**	**0-5**	3.11	0.51	0.14	0.07	0.67	0.86	1.58	0.85	0.71	0.21	0.34	0.50	0.46	0.13	0.05
	**6-10**	0.43	3.50	0.77	0.27	0.22	0.48	0.86	0.90	0.98	0.48	0.15	0.06	0.22	0.17	0.27
	**11-15**	0.05	0.42	6.56	0.37	0.47	0.28	0.61	0.72	1.18	0.73	0.61	0.06	0.11	0.07	0.37
	**16-20**	0.02	0.05	0.49	2.35	1.47	0.23	0.69	0.23	0.63	0.71	0.79	0.16	0.05	0.00	0.04
	**21-25**	0.08	0.07	0.16	0.53	2.16	0.95	1.54	0.68	1.37	0.45	1.04	0.89	0.49	0.02	0.00
	**26-30**	0.16	0.02	0.01	0.59	0.45	1.43	0.76	0.38	0.65	0.32	0.17	0.21	0.40	0.23	0.16
	**31-35**	0.38	0.28	0.84	0.18	0.80	1.03	2.32	0.58	0.92	0.49	0.51	0.34	0.79	0.12	0.23
	**36-40**	0.45	0.31	0.23	0.14	0.23	0.40	0.78	0.86	1.08	0.33	0.33	0.17	0.28	0.13	0.17
	**41-45**	0.12	0.23	0.42	0.13	0.32	0.64	0.76	0.65	1.15	0.86	0.70	0.31	0.22	0.05	0.08
	**46-50**	0.06	0.19	0.30	0.38	0.41	0.62	0.99	0.84	1.14	0.98	0.82	0.50	0.53	0.01	0.14
	**51-55**	0.07	0.14	0.26	0.34	0.49	0.55	0.84	0.47	0.77	1.25	0.97	0.51	0.33	0.07	0.20
	**56-60**	0.47	0.06	0.08	0.16	0.41	0.51	0.66	0.63	0.82	0.70	1.18	0.73	0.65	0.41	0.31
	**61-65**	0.10	0.03	0.04	0.04	0.23	0.36	0.40	0.38	0.30	0.42	0.51	0.80	0.61	0.35	0.18
	**66-70**	0.21	0.00	0.09	0.05	0.23	0.34	0.41	0.51	0.57	0.19	0.32	0.39	0.67	0.26	0.18
	**>70**	0.03	0.02	0.00	0.22	0.16	0.31	0.38	0.54	0.54	0.26	0.19	0.33	0.55	0.44	0.66

Supplementary Table S6d. Contact matrix of reported contacts weighted by IPTW, consisting of the mean number of contacts per day per participant (similar as Table S5 of Mossong et al)

Supplementary Text S1. Sample paper questionnaire in English

We showed only two contact entries in Part 3 Contact Diary. There were 100 entries in the actual paper questionnaire we provided to the participants. In the online questionnaire, a dynamic table of contact diary was provided: one additional row of contact entry was shown when participants clicked “add contact”.

Social contact survey relevant to the spread of infectious diseases

Name: ____________________________________
Participant ID: ______________________________
Assigned date: _____ _____ (DD) / _____ _____ (MM)

INTRODUCTION

Thank you for participating in our study "Population-based survey on patterns of social contacts relevant to the spread of infectious diseases in Hong Kong".

The questionnaire consists of 3 sections:

- Your demographic data
- Information about persons you usually have contact with
- Information about contacts you have made during the assigned date

- I give permission to the investigators to contact me within 12 months for potential follow-up for this study. Please provide your email address, phone number or mailing address.

Email: ____

Phone: ____

Letter: ____

PART 1: DEMOGRAPHIC DATA

1. Please provide your age OR age range:

☐ **Age: _____ Years (Please fill in your age)**

☐ **Age range (Please select your age range below):**

☐ 0-5	☐ 6-10	☐ 11-15	☐ 16-20	☐ 21-25	☐ 26-30
☐ 31-35	☐ 36-40	☐ 41-45	☐ 46-50	☐ 51-55	☐ 56-60
☐ 61-65	☐ >65	☐ Others:_____	☐ Don’t know	☐ Unwilling to answer	

2. Sex:

☐ Male ☐ Female ☐ Unwilling to answer

3. Please select your working status:

☐ Full-time ☐ Part-time ☐ Unwilling to answer

Please select your working role:

☐ Employees	☐ Employers	☐ Self-employed	☐ Students	☐ Home-makers
☐ Retired persons	☐ Others: _______	☐ Don’t know	☐ Unwilling to answer	

4. Highest level of education attained (completed):

☐ Primary or below	☐ Secondary	☐ Post-secondary
☐ Others: ________	☐ Don’t know	☐ Unwilling to answer

5. Please select to provide your individual OR household monthly income:

☐ Individual income ☐ Household income

Please provide the range of monthly income:

☐ 0-5,999	☐ 6,000-9,999	☐ 10,000-14,999	☐ 15,000-19,999	☐ 20,000-24,999
☐ 25,000-29,999	☐ 30,000-39,999	☐ 40,000-59,999	☐ 60,000-79,999	☐ 80,000-99,999
☐ ≥100,000	☐ Others:______	☐ Don’t know	☐ Unwilling to answer	

6. Place of birth:

☐ Hong Kong ☐ Mainland China ☐ Others: ______ ☐ Don’t know ☐ Unwilling to answer

7. District you are living in:

☐ Central and Western	☐ Eastern	☐ Southern	☐ Wan Chai	☐ Kowloon City
☐ Kwun Tong	☐ Sham Shui Po	☐ Yau Tsim Mong	☐ Wong Tai Sin	☐ Islands
☐ Kwai Tsing	☐ North	☐ Sai Kung	☐ Sha Tin	☐ Tai Po
☐ Tsuen Wan	☐ Tuen Mun	☐ Yuen Long	☐ Don’t know	☐ Unwilling to answer

8. Ethnicity:

☐ Chinese	☐ Indonesian	☐ Filipino	☐ White	☐ Indian
☐ Pakistani	☐ Nepalese	☐ Others: ____	☐ Don’t know	☐ Unwilling to answer

9. Are you a Hong Kong permanent resident:

☐ Yes ☐ No ☐ Unwilling to answer

PART 2: USUAL CONTACT INFORMATION

10. How many people do you live with (sleep in the place more than 4 nights per week)?

Number of persons: ☐ persons Type: ☐ Household ☐ Student residential hall ☐ Shared apartment

11. What are the age, sex and relationship to you for people who you reported in Question 10? Please list the 10 most important people if there is not enough space.

Persons who live with you	
1	**Age:** ______ years or ______ - ______ years **Sex:** ☐ Male ☐ Female<br>**Relationship:** ☐ Spouse/partner ☐ Parent ☐ Child ☐ Sibling ☐ Grandparent ☐ Grandchild<br>☐ Classmate ☐ Colleague ☐ Boy/girl friend ☐ Others ☐ No relation / Unwilling to answer
2	**Age:** ______ years or ______ - ______ years **Sex:** ☐ Male ☐ Female<br>**Relationship:** ☐ Spouse/partner ☐ Parent ☐ Child ☐ Sibling ☐ Grandparent ☐ Grandchild<br>☐ Classmate ☐ Colleague ☐ Boy/girl friend ☐ Others ☐ No relation / Unwilling to answer
3	**Age:** ______ years or ______ - ______ years **Sex:** ☐ Male ☐ Female<br>**Relationship:** ☐ Spouse/partner ☐ Parent ☐ Child ☐ Sibling ☐ Grandparent ☐ Grandchild<br>☐ Classmate ☐ Colleague ☐ Boy/girl friend ☐ Others ☐ No relation / Unwilling to answer
4	**Age:** ______ years or ______ - ______ years **Sex:** ☐ Male ☐ Female<br>**Relationship:** ☐ Spouse/partner ☐ Parent ☐ Child ☐ Sibling ☐ Grandparent ☐ Grandchild<br>☐ Classmate ☐ Colleague ☐ Boy/girl friend ☐ Others ☐ No relation / Unwilling to answer
5	**Age:** ______ years or ______ - ______ years **Sex:** ☐ Male ☐ Female<br>**Relationship:** ☐ Spouse/partner ☐ Parent ☐ Child ☐ Sibling ☐ Grandparent ☐ Grandchild<br>☐ Classmate ☐ Colleague ☐ Boy/girl friend ☐ Others ☐ No relation / Unwilling to answer
6	**Age:** ______ years or ______ - ______ years **Sex:** ☐ Male ☐ Female<br>**Relationship:** ☐ Spouse/partner ☐ Parent ☐ Child ☐ Sibling ☐ Grandparent ☐ Grandchild<br>☐ Classmate ☐ Colleague ☐ Boy/girl friend ☐ Others ☐ No relation / Unwilling to answer
7	**Age:** ______ years or ______ - ______ years **Sex:** ☐ Male ☐ Female<br>**Relationship:** ☐ Spouse/partner ☐ Parent ☐ Child ☐ Sibling ☐ Grandparent ☐ Grandchild<br>☐ Classmate ☐ Colleague ☐ Boy/girl friend ☐ Others ☐ No relation / Unwilling to answer
8	**Age:** ______ years or ______ - ______ years **Sex:** ☐ Male ☐ Female<br>**Relationship:** ☐ Spouse/partner ☐ Parent ☐ Child ☐ Sibling ☐ Grandparent ☐ Grandchild<br>☐ Classmate ☐ Colleague ☐ Boy/girl friend ☐ Others ☐ No relation / Unwilling to answer
9	**Age:** ______ years or ______ - ______ years **Sex:** ☐ Male ☐ Female<br>**Relationship:** ☐ Spouse/partner ☐ Parent ☐ Child ☐ Sibling ☐ Grandparent ☐ Grandchild<br>☐ Classmate ☐ Colleague ☐ Boy/girl friend ☐ Others ☐ No relation / Unwilling to answer
10	**Age:** ______ years or ______ - ______ years **Sex:** ☐ Male ☐ Female<br>**Relationship:** ☐ Spouse/partner ☐ Parent ☐ Child ☐ Sibling ☐ Grandparent ☐ Grandchild<br>☐ Classmate ☐ Colleague ☐ Boy/girl friend ☐ Others ☐ No relation / Unwilling to answer

PART 3: CONTACT DIARY

INSTRUCTIONS

1) Record in the contact diary **every person** you have contact with on your assigned day, regardless of whether the contact was long or short, and whether you know the person or not.
2) A contact is defined as:
   - EITHER **skin-to-skin contact** (physical contact, such as a handshake, hug, kiss)
   - OR **face-to-face conversation** with three or more words exchanged, within 1-2 meters (non-physical contact). Ignore conversations made over phones or on computers.
3) If you contact the same person several times during the assigned day, only record him/her once, and record the total time you spent with that person over the entire day. So each person you meet during the day and have contact with should have one line in the diary.

12. The date you fill in the questionnaire:

_____ (DD) / _____ (MM)

13. For the purposes of this study, the day starts at 5 a.m. on the morning and ends at 5 a.m. the next morning. Provide contact details by filling in the table:

#	Name or description	Contact demographics	Contact details	Record time	How often do you have contact with this person?
1		**Age:** _____ or _____ – _____ years<br>**Sex:** ☐ Male ☐ Female<br>**Member of :**<br>☐ Home ☐ Student hall<br>☐ School ☐ Shared apartment<br>☐ Work ☐ Others	**Contact type:** ☐ Skin-to-skin ☐ Non-physical<br>**Total time:**<br>☐ <5 mins<br>☐ 5-14 mins<br>☐ 15-59 mins<br>☐ 1-4 hrs<br>☐ > 4 hrs<br>**Where:**<br>☐ House<br>☐ Work<br>☐ School<br>☐ Transport<br>☐ Others	___: ___	**How often in general:**<br>☐ Daily or almost daily<br>☐ Once or twice a week<br>☐ Once or twice a month<br>☐ Less than once a month<br>☐ Never met before
2		**Age:** _____ or _____ – _____ years<br>**Sex:** ☐ Male ☐ Female<br>**Member of :**<br>☐ Home ☐ Student hall<br>☐ School ☐ Shared apartment<br>☐ Work ☐ Others	**Contact type:** ☐ Skin-to-skin ☐ Non-physical<br>**Total time:**<br>☐ <5 mins<br>☐ 5-14 mins<br>☐ 15-59 mins<br>☐ 1-4 hrs<br>☐ > 4 hrs<br>**Where:**<br>☐ House<br>☐ Work<br>☐ School<br>☐ Transport<br>☐ Others	___: ___	**How often in general:**<br>☐ Daily or almost daily<br>☐ Once or twice a week<br>☐ Once or twice a month<br>☐ Less than once a month<br>☐ Never met before

Before submitting the contact diary, please make sure you have not left out any contacts.

We do ask you to include every contact you had, but if you were unable to include every single contact (for instance, because you work in a shop and have a large number of contacts in a day), please could you indicate this in Question 14?

14. Have you included all contacts in Question 13?

☐ Yes
☐ No, approximately how many people you have left out: ________________________________

--**END**--

Once again, thank you for your participation.

References

1 Wu, J. T. *et al.* Inferring Influenza Infection Attack Rate from Seroprevalence Data. *PLOS Pathogens* **10**, e1004054, doi:10.1371/journal.ppat.1004054 (2014).

2 Mossong, J. *et al.* Social Contacts and Mixing Patterns Relevant to the Spread of Infectious Diseases. *PLOS Medicine* **5**, e74, doi:10.1371/journal.pmed.0050074 (2008).
